# Thermally Tunable Acoustic Beam Splitter Based on Poly(vinyl alcohol) Poly(N-isopropylacrylamide) Hydrogel

**DOI:** 10.3390/gels7030140

**Published:** 2021-09-13

**Authors:** Yuqi Jin, Mi Zhou, Tae-Youl Choi, Arup Neogi

**Affiliations:** 1 Department of Physics, University of North Texas, Denton, TX 76203, USA; yuqijin@my.unt.edu; 2 Department of Mechanical Engineering, University of North Texas, Denton, TX 76207, USA; tae-youl.choi@unt.edu; 3 Center for Agile and Adaptive Additive Manufacturing, University of North Texas, Denton, TX 76207, USA; 4 Department of Electrical Engineering, University of North Texas, Denton, TX 76207, USA; mizhou2@my.unt.edu

**Keywords:** hydrogel, beam splitting, thermal tunable, transparent acoustic impedance

## Abstract

In this study, we demonstrated a thermally tunable acoustic beam splitter using a poly(vinyl alcohol) poly(N-isopropylacrylamide) hydrogel (PVA-pNIPAM). The nature of PVA-pNIPAM hydrogel offers exceptional temperature-dependent physical properties due to its phase transition around its lower critical solution temperature. The acoustic impedance of the hydrogel can be tuned below, above, or matched to that of water by changing the environmental temperature. An acoustic wave propagating in water can be split into transmitted and reflected components by the PVA-pNIPAM hydrogel slab on varying its angle of incidence. The intensity ratio between the reflected and the transmitted componence can be adjusted by tuning the temperature of the medium. The acoustic beam can be entirely reflected at a temperature corresponding to the matched impedance between hydrogel and water. The beam-splitting behavior was observed for acoustic waves from both a monochromatic wave and broadband pulse source. In addition, the phase of beam split pulses can be reversed by selecting the hydrogel’s operating temperature.

## 1. Introduction

The anomalous phase transition in certain liquid or hybrid hydrogel composites can be induced by varying the environmental pH condition [1] or temperature [2]. The thermal responsive hydrogels commonly transform from a hydrophilic state to a hydrophobic state around its lower critical solution point (LCST). The water–polymer crosslinks break, which then form into polymer–polymer links [3]. The anomalous phase transition of thermally tunable hydrogels opens a great potential in drug-delivering [4], sensing [5], and micro-robotics [6] fields. Bulk poly(N-isopropylacrylamide) (pNIPAM) composite is one of the commonly studied thermal sensitive hydrogels with a sharp transition in volumetric and mechanical properties around its LCST [7]. The dramatic change in the physical properties was observed in terms of physical volume [8], static elasticity [9], dynamic elasticity [10], density [11], and swelling/deswelling ratios [12]. The reported behaviors and characterizations have resulted in the development of practical applications, such as micropumps [13], small-scale surgery [14], acoustic lens [15], and even 4D additive manufacturing products [16].

Based on the similarity in the physical properties between PVA-pNIPAM hydrogel and water, we investigate the feasibility of controlling the underwater acoustic wave propagation by designing an acoustic beam splitter for active acoustic systems. Acoustic beam splitters were usually realized by 2D phononic crystal structure-based devices [17]. The specific waveguide arrangement in the array of scatters can separate acoustic waves into multiple channels [18]. The transmission through phononic crystal waveguides is relatively low due to reflection and transmission losses due to scattering. A bulk phononic crystal can also act as a beam splitter if its high transmission band operates at wavelengths comparable to its periodicity [19]. However, due to the difficulty in fabricating 3D phononic crystals, the commonly used 2D phononic crystal distorts the acoustic beam profile along the third axis. Hence, a relatively homogeneous material beam splitter with tunability is necessary for future phononics devices to realize acoustic systems similar to optical systems and electric circuits in photonics and electronics.

We previously reported the sound velocity, density, and dynamic bulk modulus in PVA-pNIPAM hydrogel [10,20]. These properties are critical in steering the propagation of longitudinal sound waves. Hence, we realized that the acoustic impedance Z could be correspondingly varied in PVA-pNIPAM hydrogel with respect to the sound velocity c and density ρ by changing the environmental temperature. According to the definition: Z=ρc, the acoustic impedance of PVA-pNIPAM hydrogel can approximately match that of water around its LCST. The feature was numerically simulated and characterized by experimental approaches in this study. It was observed that the PVA-pNIPAM hydrogel’s acoustic impedance could be lower and higher than water at a temperature below and above its LCST, respectively. A novel active thermally tunable acoustic beam splitter was designed with a slab of PVA-pNIPAM hydrogel in water ambient using numerical approaches. At a critical incident angle, the acoustic ray can be separated into reflected and refracted components. The propagating beam can have varying transmissivity at specific operating temperatures induced by the impedance mismatch between the hydrogel slab within the ambient water. At the impedance-matched temperature, the absence of the reflection ray led to a significant increase in the transmission intensity. The tunable beam splitter can operate with monochromatic acoustic and broadband pulse sources in the frequency and time domains. Additionally, it also observed that the sign of the impedance mismatch ($Z-Z_{\mathrm{water}}$) dictated the phase of the reflected component. The phase of the reflected pulse envelopes can be reversed by a change in the impedance mismatch around LCST due to the volumetric phase transition temperature of the hydrogel.

## 2. Results and Discussion

**Figure 1:**
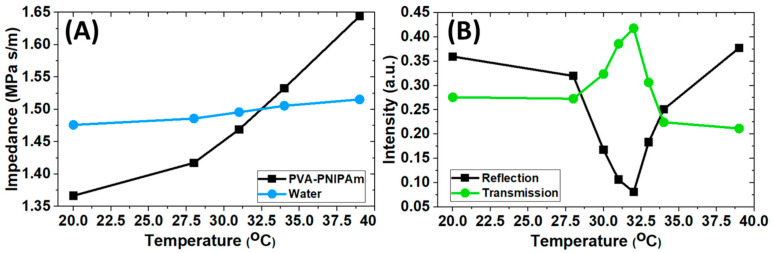
(**A**) Experimentally characterized temperature-dependent acoustic impedance of PVA-pNIPAM hydrogel and the comparison with water. (**B**) Experimental transmission and reflection behavior of the hydrogel in water ambient at varying operating temperatures.

To obtain the temperature-dependent acoustic impedance of PVA-pNIPAM hydrogel, we initially performed temperature-dependent acoustic transmission measurements in water ambient using a bistatic setup. Figure 1A shows the acoustic impedance in our bulk PVA-pNIPAM hydrogel. The experimental values were obtained from the measured speed of sound and density by the methods demonstrated in our previous study [20]. The temperature range investigated was from 20 °C to 39 °C. The two temperature points are typically considered stable hydrophilic and hydrophobic states below and above the LCST, respectively. The temperature-dependent acoustic impedance in water is depicted in Figure 1A for comparison. In this narrow temperature range, deionized water shows weak temperature dependence. The variation of the impedance of water is contributed by the increase in water’s sound velocity temperature. In the same temperature range, the acoustic impedance of bulk PVA-pNIPAM hydrogel increased sharply from 28 °C to 39 °C. This increase is about 35%, contributed to by the increase in both PVA-pNIPAM hydrogel’s speed of sound and density. At around 32 °C, a crossing point can be found between temperature-dependent acoustic impedance lines in PVA-pNIPAM hydrogel and water, indicating the acoustic impedance of the hydrogel can be matched with DI water to achieve acoustic transparency. Our previous experimental observation [15] shows that the heating rate of hydrogel media does not deviate its acoustic properties within the specific temperature range of which the experiments have considered in the present work. The temperature dependent impedance behavior of the hydrogel is reversible if the ambient temperature of the DI water in which the hydrogel medium is immersed is changed within a range that does not any modify its density leading.

To verify the temperature-dependent impedance, an additional temperature-dependent transmission and reflection experiment was performed using a bistatic setup. A hydrogel disc was aligned in between two identical ultrasound planar transducers. The surfaces of the disc sample were parallel to the transducer surfaces to have normal incidence. One of the transducers emitted an acoustic pulse propagating in water ambient to the hydrogel in the experiment. The reflection signals were collected at the emission source transducer. Another transducer acquired the signal transmitted through the hydrogel sample. In Figure 1B, the plot shows the normalized (by the emission source intensity) signal intensity of the collected reflection and transmission at different temperatures. From room temperature to 28 °C, we observed a reduction in the reflection intensity as the hydrogel’s acoustic impedance increasingly approached the impedance of the ambient water. However, the transmission intensity we measured did not have a corresponding rise. As the total emitted acoustic source energy was constant, we conclude that the absorption within the hydrogel increased from 20 °C to 28 °C. From 28 °C to 32 °C, a sharp rise in the transmission through the hydrogel sample was observed. It is associated with a decrease in reflection intensity. At 32 °C, the transmission intensity reached its maximum with the minimum reflection measured below 8% of the source intensity.

Around 32 °C, the hydrogel sample can be considered as acoustically transparent. As the environmental temperature further increased, the acoustic impedance of the hydrogel sample kept increasing after passing through the matching point with ambient water. The reflection (transmission) intensity increased (decreased) again due to the increasingly enhanced impedance mismatch between the hydrogel sample and ambient water from 32 °C to 34 °C. Beyond the phase transition temperature (34 °C to 39 °C), the transmission through the hydrogel was stable. From this experiment, we verified that hydrogel could be approximately acoustically transparent around 32 °C. And the hydrogel can provide a comparable amount of reflection and transmission intensity in the temperature ranges 20 °C to 30 °C and 34 °C to 39 °C respectively. Therefore, using a bulk PVA-pNIPAM hydrogel to make a tunable acoustic beam splitter with a material-dependent transparency point.

**Figure 2:**
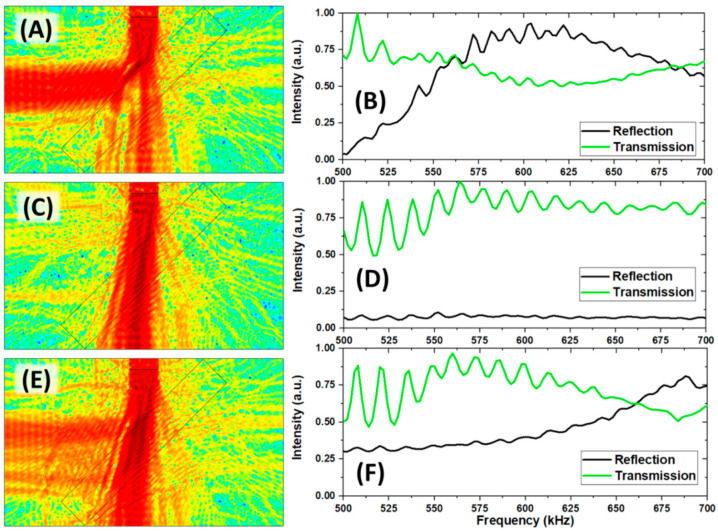
(**A**) Numerical simulation of acoustic separation by hydrogel in water presented by acoustic intensity field at 600 kHz and 20 °C. (**B**) Simulated frequency spectra of the separated reflection and transmission at 20 °C. (**C**) Numerical simulation of acoustic separation at 600 kHz and 32 °C. (**D**) Simulated frequency spectra of the separated reflection and transmission at 32 °C. (**E**) Numerical simulation of acoustic separation at 600 kHz and 39 °C. (**F**) Simulated frequency spectra of the separated reflection and transmission at 39 °C.

Based on the above experimental verification, we proposed a simple thermally tunable acoustic beam splitter design by following numerical simulations with input experimental measured temperature-dependent properties of PVA-pNIPAM hydrogel. As Figure 2A,C,E illustrate, a PVA-pNIPAM hydrogel slab was placed in water ambient along a 45° angle. An acoustic transducer was placed at the upper edge of the water tank, excited single-frequency continuous wave at 0° vertically, 45° incidence respective to the PVA-PNIPAm hydrogel slab. Figure 2A,C,E showed the sound intensity field maps at 20 °C, 32 °C, and 39 °C at 600 kHz. The beam-splitting behavior of the hydrogel slab was temperature-dependent. The acoustic beam was separated into reflection and transmission beams with different intensities at 20 °C (Figure 2A) and 39 °C (Figure 2E). At impedance-matched temperature 32 °C (Figure 2C), a very limited amount of reflection energy was observed on the left side of the hydrogel slab.

The broader frequency range was studied for the three cases, and the results were plotted as normalized frequency spectra in Figure 2B,D,F. In these figures, the lines in each subplot were normalized by their maximum values. In Figure 2B,F, we observed a broadening of the transmission spectra at the two different temperatures. The crossing point between the transmission intensity and reflection intensity lines had a blue shift due to increased interacting wavelength in the hydrogel slab. At an elevated temperature, the increase in the speed of sound within the hydrogel led to the change in the reflectivity peak. The temperature-dependent elastic constant of the medium modifies the hydrogel’s refractive index, leading to the reflection of the wavelength of the waves at a lower frequency. At 20 °C (39 °C), a part of the incident wave propagated inside the slab and dissipated energy from around 550 kHz (625 kHz). Taking advantage of the blue shift on the frequency spectra between 20 °C and 39 °C, the thermally adjustable intensity ratio between the separated transmission and reflection rays can be obtained at a certain operating frequency.

On the other hand, the spectra in Figure 2D showed low reflection over the entire studied frequency range at the impedance-matched temperature of 32 °C. Based on the extent of impedance mismatch, the intensity of the transmission rays was expected to be higher than we observed.

**Figure 3:**
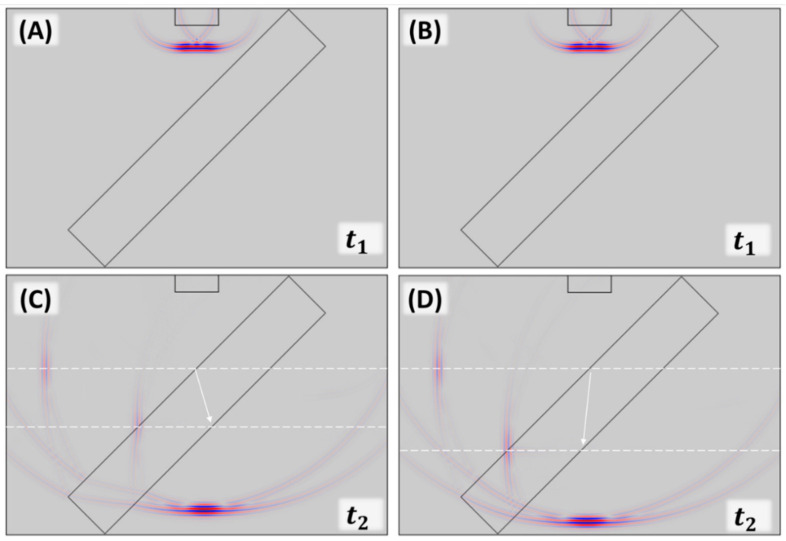
(**A**) Time-domain simulation of sound pressure field showing the incident pulse at 20 °C. (**B**) Time-domain simulation of sound pressure field showing the identical incident pulse from (**A**) at 39 °C and $t_{1}$. (**C**) Separated pulses at 20 °C and $t_{2}$. (**D**) Separated pulses at 39 °C and $t_{2}$. The white dash lines indicated the vertical position of the pulses’ center. The white arrows referred to the propagation paths in the hydrogel slab.

**Figure 4:**
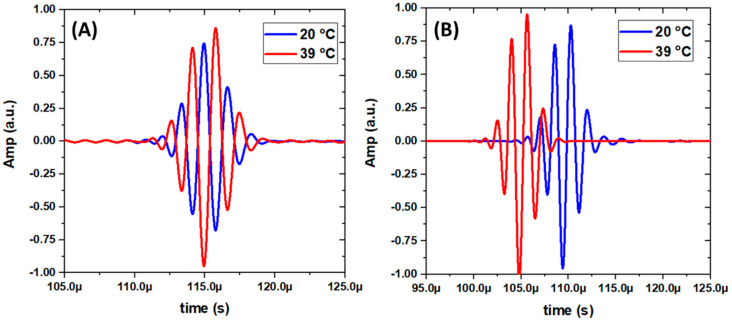
Measured acoustic reflection signals obtained from the setup shown in Figure 3. (**A**) Reflections. (**B**) Transmissions. Measured acoustic transmission signals obtained from the setup shown in Figure 3.

**Figure 5:**
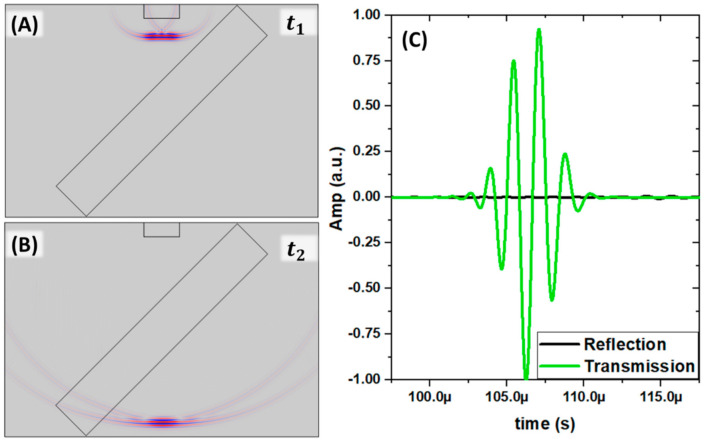
(**A**) Time-domain simulation of sound pressure field showing the incident pulse at 32 °C (**B**) Separated pulses at 32 °C and $t_{2}$. (**C**) Measured acoustic reflection and transmission signals from (**A**,**B**).

In many acoustic practical applications, the acoustic sources are typically broadband pulse to have time information. Hence, we also investigated the effectiveness of the impedance mismatched principle on a broadband acoustic pulse by changing the operating temperature. Figure 3, Figure 4 and Figure 5 show the beam splitter operation in the time-domain with a broadband pulse source. Figure 3 illustrated the numerical model of our time-domain numerical experiments at 20 °C and 39 °C. At t_1_, the identical broadband pulse emitted by the transducers propagated to the hydrogel slab at 20 °C and 39 °C. At t_2_, the source pulse was separated into three pulses. The transmission pulse showed around the lower horizontal edge of the water tank. On the left side of the water tank, two reflection signals produced by the hydrogel slab occurred at the front and back edges at the water/hydrogel interface and the hydrogel/water interfaces due to impedance mismatch. With the close-to-water impedance, the two reflections can have comparable intensity. As the white dash lines indicated, the spatial positions of the first reflection signals were observed as expected. However, the second reflections that occurred from the back hydrogel surface have a vertically spatial offset. The offset was caused by the difference in the hydrogel’s speed of sound at 20 °C (lower than water) and 39 °C (higher than water). The index of refraction n in the hydrogel from the water ambient has a significant difference between n > 1 at 20 °C and 0 < n < 1 at 39 °C, which led to the different wave paths shown by the white arrows.

Furthermore, the acoustic impedance of the hydrogel can be tuned from lower-than-water to higher-than-water from 20 °C to 39 °C, as Figure 1 illustrated. The PVA-pNIPAM hydrogel’s temperature-dependent impedance behavior can introduce phase reversal of the acoustic wave in water ambient, a unique functionality. Figure 4 shows the time domain signals collected from the numerical model which is illustrated in Figure 3. In general, the separated pulses had higher amplitude at 39 °C than 20 °C as the hydrogel’s higher density at 39 °C induced less energy dissipation. In Figure 4A, a phase reversal behavior from the acoustic waves reflected from the gel at 20 °C and 39 °C is shown. The behavior occurred due to the change of PVA-pNIPAM hydrogel from a softer medium-to-hard medium (from 20 °C to 39 °C) comparing with ambient water. In Figure 4B, the transmission obtained at 20 °C and 39 °C does not exhibit any phase reversal. Hence, the proposed PVA-pNIPAM hydrogel slab can offer matched or mismatched phases of separated pulse envelopes depending on the operating temperature. The novel temperature-dependent phase modulation behaviors of acoustic broadband pulse sources have never been demonstrated in the existing literature. The simulated animations are in Supplementary Materials.

Figure 5 shows the time-domain pulse-sourced simulation results at the impedance-matched temperature 32 °C. At t_1_ (subfigure A), the broadband pulse emitted by the transducer transmitted through the hydrogel slab without creating a visible reflection (Figure 5B). We plotted the collected signals from the lower edge and left side edge on the water tank in Subfigure C. The lines were normalized with an identical factor equal to the absolute maximum amplitude of the transmission signal. The maximum amplitude collected on the reflection side was only about 3% of the transmission side. It further confirms that the PVA-PNIPAm slab can be transparent at 32 °C to acoustic pulse.

The existing design of acoustic beam splitters includes phononic crystals [21], topological metamaterials [22], asymmetric artificial meta-surfaces [23]. These designs were complex and introduced limitations due to fabrication, a narrow operating band, strong frequency dispersion, and nonreciprocity. In addition, at a certain operating frequency, the existing approaches cannot modify the intensity ratio between the separated acoustic components. In the existing literature, another in-air 3-port active non-reciprocal device [24] could achieve tunable beam separation with the changing speed in the circulating fluid. However, for underwater acoustics, an additional powered pumping source also introduces certain complexity in design. Especially in ultrasound applications, the most common pumping sources have comparable or larger sizes compared to the operating wavelength in water. Using the temperature-dependent acoustic impedance of PVA-pNIPAM hydrogel, our designed thermally responsible hydrogel slab separates the source beam into reflection and transmission with tunable intensity ratio and transparency function. It is not limited to a narrow frequency range or any active external pumping source. Comparing with the existing solution of an acoustic separator or splitters, the advantages of our design were tunability, wider operating frequency, flexibility to scale up/down with very different operating frequencies, easy manufacturing, and reciprocal propagation.

Based on previous reports [11,20] of the acoustic properties of pNIPAM hydrogel and PVA-pNIPAM hydrogel, sound propagates slower in PVA-pNIPAM hydrogel in comparison to pNIPAM hydrogel. The slower sound velocity in PVA-pNIPAM hydrogel increased its contrast in acoustic impedance mismatch with the ambient water at various temperatures. To realize a tunable beam separator, using solely pNIPAM hydrogel would reduce the contrast in its sound wave propagation characteristics with water at different temperatures.

Furthermore, the acoustic impedance of the hydrogel can be tuned to be transparent in water, which is a special function and never was demonstrated before. In electronics, the transparent impedance of an extra component can be achieved by applying a wavelength phase delay [25]. However, the methods can only perform well with the monochromatic source but not broadband pulse. Our proposed hydrogel slab impedance can be physically tuned to match the ambient water to overcome the limitation of using pulse in conventional techniques if the temperature control can be regulated. Furthermore, microwave or other electromagnetic wave-based heating methods can be applied to the hydrogel for rapid heating in terms of uniformity and operation speed. Alternatively, by using a thinner slice of the hydrogel slab (with a reduced volume), the beam splitter can be operated at a significantly higher operating frequency. The size deviation that occurs due to the deswelling effect cannot change the behavior of the temperature-dependent impedance hydrogel beam splitter, as long as the thickness of the slab is greater than one operating wavelength. Hence, the minimum thickness after deswelling can be calculated ($d_{\min}>c_{39\;{}^{\circ}C}/f$) based on the operating frequency f and the speed of sound $c_{39\;{}^{\circ}C}$ reported in the previous study [20]. To avoid experimental error, the minimum sample thickness can be optimized by considering the initial volume of the hydrogel at a temperature at 39 °C.

## 3. Conclusions

In summary, we designed a novel thermal tunable acoustic beam separator with an acoustically transparent function by thermally phase transitive bulk PVA-pNIPAM hydrogel. The mechanism of the tunable separator was based on the temperature acoustic impedance of the PVA-pNIPAM hydrogel. From room temperature to 39 °C, the acoustic impedance of the hydrogel can be changed dramatically from lower than water, matched with water, and higher than water. The temperature dependence on the impedance of the hydrogel was experimentally characterized. We designed the thermal tunable hydrogel acoustic separator numerically based on the characterized acoustic properties of the PVA-pNIPAM hydrogel. Then, the beam-splitting behavior was experimentally demonstrated by measuring the temperature-dependent transmission/reflection intensity ratio of PVA-pNIPAM hydrogel in water ambient. The designed separator can split the incident beam into reflected and refracted beams in a frequency-domain simulation with a single-frequency continuous wave source. The intensity of the two components can be thermally tunable. At the impedance-matched temperature between hydrogel and water, the separator can be transparent without any reflected component. In the time-domain simulation with a broadband pulse source, in addition to the beam spitting and transparent functionalities, the hydrogel separator can split the source pulse into two resulting pulses with the same or reversed phase, dependent on the operating temperature.

## 4. Materials and Methods

### 4.1. PVA-pNIPAM Hydrogel

The PVA-pNIPAM hydrogel composites were prepared by dissolving NIPAM monomer (TCI Chemicals, Tokyo, Japan) and N’-methyene-biacrylamide crosslinker (BIS; Polysciences Inc., Warrington, PA, USA) in DI water. The NIPAM:BIS:DI water weight ratio was 0.1:0.02:0.85 Poly (Vinyl Alcohol) (PVA) (Polysciences Inc., Warrington, PA, USA) was added to the monomer solution by weight ratio 0.03 of the total weight. The PVA molecular weight was about 25,000 and is 98% hydrolyzed. To dissolve PVA, the mixture was heated at 50 °C under stirring for more than 24 h. After complete dissolution, the solution was cooled down using an ice bath under N_2_ for more than an hour to remove oxygen. To initiate polymerization, 0.03 ammonium persulfate (Sigma-Aldrich, St. Louis, MO, USA) and 0.015 tetramethylethylenediamine (TEMED) (Sigma-Aldrich, St. Louis, MO, USA) were used by the weight ratio of the total mixture at room temperature. The time duration for the polymerization was about 6 h. The solidified hydrogel was then placed in a deionized water bath for more than two days to further swell. As PVA is soluble in water, replacing the unreacted PVA water solution with DI water in the hydrogel is essential. In the clean DI-water, the immersed hydrogel was heated and cooled to remove water-soluble impurities. This water solution was replaced with another round of clean DI water. The process was repeated five times to clean the hydrogel. Finally, the processed bulk hydrogel was ready to dehydrate and hydrate for being used reversibly. Under the polymerization conditions (10–15% of total gel concentration), the reaction went to 90–96% completion.

### 4.2. Experimental Setup

**Figure 6:**
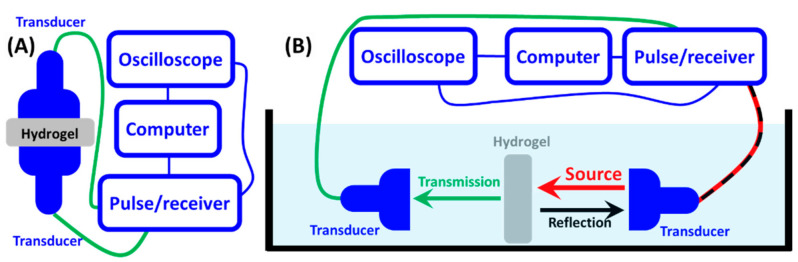
(**A**) Bistatic speed of sound experimental measurement for determining the temperature-dependent acoustic impedance of the hydrogel. (**B**) Temperature-dependent bistatic transmission and reflection experiments of hydrogel in water.

The temperature-dependent acoustic impedance of PVA-pNIPAM hydrogel was obtained by measuring its sound velocity and density. Density measurement was performed using a pycnometer with disc-shape samples. In the speed of sound measurements (Figure 6A), the JSR (Pittsford, NY, USA) DPR300 Pulser/Receiver (signal source and collector) and Tektronix (Beaverton, OR, USA) MDO 3024 oscilloscope were used. Olympus (Shinjuku City, Tokyo, Japan) pulse immersion transducers (at 0.5 MHz-center-frequency with 1-inch negative spike) were used in this experiment. Bistatic experimental setup with a set of pulser and receiver at the two separated ports was used. Besides 20 °C (room temperature), specimens needed to be heated up with a heating lamp to 28 °C, 31 °C, 34 °C, and 39 °C for the time-of-flight measurements. The hydrogel samples’ temperature was monitored by thermocouples (4 Ω, 0.5 mm) connected to N.I. C-Series temperature input module with NI (Austin, TX, USA) USB compact DAQ chassis to read the temperature on the computer for an average temperature value.

The four thermocouples were inserted into the hydrogel disc samples. At each temperature point, three specimens with all different thicknesses were measured. For the pulse repetition frequency measurements, the DPR300 pulser sent a pulse every 2 ms. The total time range was set to 8 ms on an oscilloscope to record three waves on a single specimen and minimize the experimental error. The speed of sound in PVA-pNIPAM hydrogel was determined from the ratio of specimen thickness and travel time of the sound wave. The bistatic experiment was moved to a deionized water-infilled tank for the temperature-dependent transmission and reflection measurements (Figure 6B).

The entire tank of water was heated with a strip-type resistive heater. The hydrogel sample was placed between the two parallel facets of the transducers. The distance between the hydrogel sample and each transducer was about 12 mm. In the acoustic measurements, the emission transducer sent a pulse while another transducer on the opposite side recorded the transmission signals. In the data analysis, the emission pulse intensity was used as a normalization factor. The emission pulse intensity was measured with the same bistatic experimental setup in the water tank without the PVA-pNIPAM hydrogel sample. The emission pulse intensity in water was temperature-independent in our studied temperature range, based on our experimental observation.

### 4.3. Models in Numerical Simulation

The numerical simulations were performed by finite elements analysis (FEA) based COMSOL-Multiphysics software (Burlington, MA, USA) in the pressure acoustic module. The 25 mm wide transducer was placed on the upper edge of a 250 mm wide, 150 mm height water. The outer edges on the tank were set to an impedance matched condition to infilled water for avoiding additional energy reflection from the edges of the tank. A 180 mm wide and 30 mm thick PVA-pNIPAM hydrogel slab was placed on the center of the tank at a 45° inclined angle. The source transducer emitted a monochromatic plane wave with 0° inclined angle respected to the normal direction at 10^−6^ Pa pressure amplitude. The frequency sweep was performed in the frequency domain study from 500 kHz to 700 kHz with 1 kHz interval steps. The reflection and transmission intensity spectra were measured on the water tank’s left side and bottom edges. The averaged mesh element size was 0.4515 mm with a minimum element size of 0.2946 mm. For the time domain simulations, the time explicit pressure acoustic module was used with time-dependent study. The width of the water tank was reduced to 220 mm for a shorter calculation time. The source was a pulse function expressed as $x\left(t\right)=\sin\left(2{\pi f}_{0}t\right)e^{-f_{0}{\left(t-{\delta T}_{0}\right)}^{2}}$. The $T_{0}$ was defined as $1/f_{0}$, where $f_{0}$ was the center frequency of the pulse (600 kHz). The averaged mesh element size in the time domain simulation was 0.4506 mm with a minimum element size of 0.2255 mm. The recorded transmission and reflection pulses were collected at the water tank’s left side and bottom edges. The temperature-dependent physical properties of water were selected from the built-in library in the simulation software. The temperature-dependent speed of sound and density of PVA-pNIPAM hydrogel was obtained from the experimental characterization by the methods described above. At 20 °C, 32 °C, and 39 °C, the speed of sound values was 1342 m/s, 1455 m/s, and 1554 m/s, and the density values were 1018 Kg/m^3^, 1014 Kg/m^3^, and 1058 Kg/m^3^ pectively.

## Data Availability

Data is available from the corresponding author.

## Supplementary Materials

The following are available online at https://www.mdpi.com/article/10.3390/gels7030140/s1, Figure S1: time domain wave propagations.
