# Supplementary figures and images for: Thermally Tunable Acoustic Beam Splitter Based on Poly(vinyl alcohol) Poly(N-isopropylacrylamide) Hydrogel
Source: Gels. 2021 Sep 13;7(3):140. doi: 10.3390/gels7030140 (PMC8482244; doi:10.3390/gels7030140)

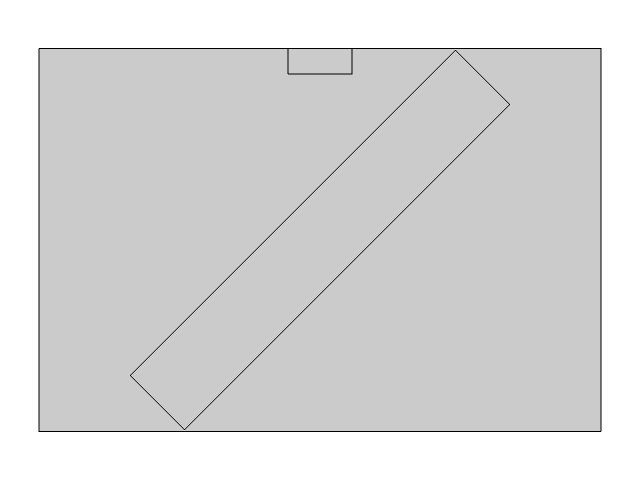

Supplement: Supplementary file 1 [file gels-07-00140-s001.zip › 20C 45deg.gif]

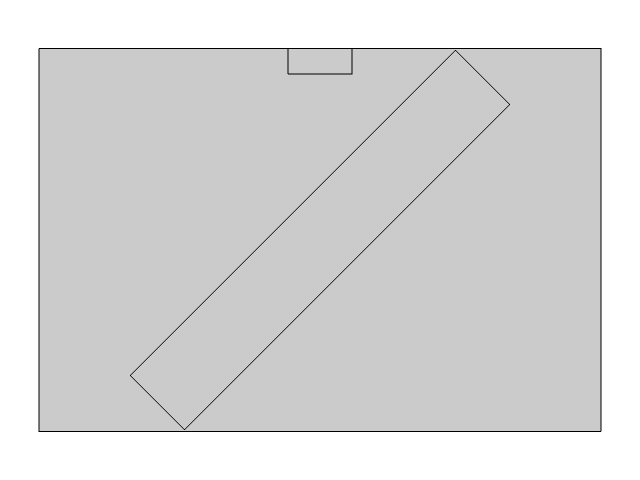

Supplement: Supplementary file 1 [file gels-07-00140-s001.zip › 31C 45deg.gif]

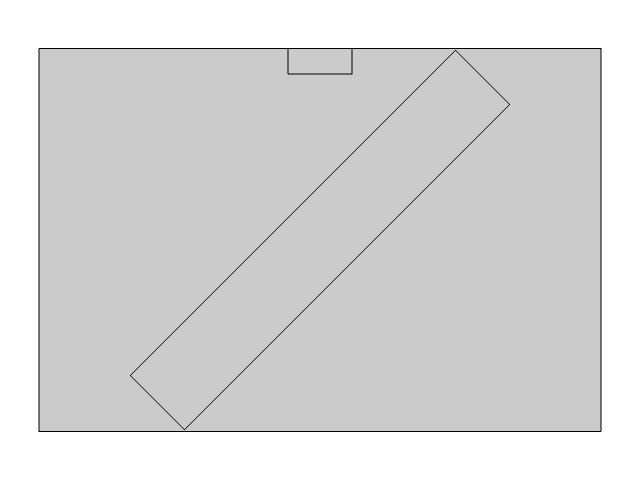

Supplement: Supplementary file 1 [file gels-07-00140-s001.zip › 39C 45deg.gif]
